# Changes of brain structure and structural covariance networks in Parkinson’s disease with different sides of onset

**DOI:** 10.3389/fnagi.2025.1564754

**Published:** 2025-04-15

**Authors:** Tianqi Xu, Zhihuai Deng, Yinhui Yu, Wenchao Duan, Zeyu Ma, Haoran Liu, Lianling Li, Moxuan Zhang, Siyu Zhou, Pengda Yang, Xueyan Qin, Zhenyu Zhang, Fangang Meng, Yuchen Ji

**Affiliations:** ^1^Department of Neurosurgery, The First Affiliated Hospital of Zhengzhou University, Zhengzhou, Henan, China; ^2^Beijing Neurosurgical Institute, Capital Medical University, Beijing, China; ^3^Yantai Affiliated Hospital of Binzhou Medical University, Yantai, Shandong, China; ^4^Department of Neurosurgery, Beijing Tiantan Hospital, Capital Medical University, Beijing, China

**Keywords:** Parkinson’s disease, asymmetry, side-of-onset, surface-based morphometry, structural covariance networks, cortical thickness, cortical surface area

## Abstract

**Background:**

Parkinson’s disease (PD) typically presents with unilateral symptoms in early stages, starting on one side and progressing, with the onset side showing more severe motor symptoms even after bilateralization. This asymmetry may reflect complex interactions among multiple brain regions and their network connections. In this study, we aimed to use surface-based morphometry (SBM) and structural covariance networks (SCNs) to investigate the differences in brain structure and network characteristics between patients with left-onset PD (LPD) and right-onset PD (RPD).

**Methods:**

A total of 51 LPD and 49 RPD patients were recruited. Clinical assessments included the Unified Parkinson’s Disease Rating Scale motor section, Hoehn and Yahr stage, Mini-Mental State Examination, Parkinson’s Disease Questionnaire, and Beck Depression Inventory. All participants underwent 3 T structural MRI. FreeSurfer was used to perform vertex-wise comparisons of cortical surface area (CSA) and cortical thickness (CT), whereas the Brain Connectivity Toolbox was implemented to construct and analyze the structural covariance networks.

**Results:**

In patients with LPD, we found reduced CSA in the right supramarginal gyrus (SMG), right precuneus (PCUN), left inferior parietal lobule (IPL), and left lingual gyrus (LING) compared to RPD, while no significant differences in CT were found between the two groups. The CSA of the right PCUN showed a significant positive correlation with MMSE score in LPD patients. In our SCNs analysis, LPD patients exhibited increased normalized characteristic path length and decreased small-world index in CSA-based networks, while in CT-based networks, they showed increased small-world index and global efficiency compared to RPD. No significant differences in nodal characteristics were observed in either CSA-based or CT-based networks between the two groups.

**Conclusion:**

In patients with LPD, reductions in CSA observed in the right PCUN, right SMG, left IPL, and left LING may be associated with cognitive impairments and hallucinations among non-motor symptoms of PD. Additionally, the SCNs of LPD and RPD patients show significant differences in global topology, but regional node characteristics do not reflect lateralization differences. These findings offer new insights into the mechanisms of symptom lateralization in PD from the perspective of brain regional structure and network topology.

## Introduction

Parkinson’s disease (PD) is the second most common neurodegenerative disorder after Alzheimer’s disease, characterized by a spectrum of progressive motor and non-motor symptoms (Aarsland et al., 2021). The pathophysiology of PD is complex and not yet fully understood. However, one prominent clinical feature of PD is the asymmetry of motor symptoms, which typically begin on one side of the body and later progress to the other (Wang et al., 2015). The onset side, also known as the symptomatic dominant side, often displays more severe motor symptoms even when the disease becomes clinically bilateral (Djaldetti et al., 2006). Unlike the symmetric presentation of multiple-system atrophy and progressive supranuclear palsy in their classic forms, this asymmetry in PD may reflect its unique heterogeneity and provides valuable insights into its progression mechanisms (Postuma et al., 2015; Wang Z. et al., 2016; Miki et al., 2021).

Numerous studies have found that the side of onset of motor symptoms in PD might influence their clinical characteristics and the progression of non-motor symptoms. For instance, left-onset PD (LPD) often show poorer visual memory and visuospatial impairments (Amick et al., 2006; Verreyt et al., 2011), more frequent hallucinations (Stavitsky et al., 2008), and a higher prevalence of rapid eye movement sleep behavior disorder (Baumann et al., 2014). In contrast, right-onset PD (RPD) is associated with poorer verbal memory and language task impairments (Amick et al., 2006; Verreyt et al., 2011), apathy (Harris et al., 2013), and a higher risk of impulse control behaviors (Phillipps et al., 2020). These clinical differences highlight the potential impact of PD lateralization on non-motor symptoms, possibly reflecting underlying brain structure variations. However, the mechanisms involved in PD asymmetry have not yet been elucidated.

Structural magnetic resonance imaging (MRI) studies offer preliminary evidence for the lateralization of PD. For example, LPD patients show reduced gray matter volume in the right middle frontal gyrus and precuneus (PCUN), which are closely linked to visuospatial memory impairment (Lee et al., 2015). Additionally, LPD patients show cortical thinning in motor-related areas of the left hemisphere (Kim et al., 2014). Conversely, studies on cortical complexity in RPD patients have revealed decreased mean fractal dimension and mean sulcal depth in the left superior temporal sulcus compared to LPD patients (Zhang et al., 2021). Although these findings provide some insight into brain structure changes related to PD lateralization, limited research has focused on cortical surface area (CSA) and cortical thickness (CT). Surface-based morphometry (SBM) tools such as FreeSurfer can accurately quantify CSA and CT (Goto et al., 2022). CSA indicates the unfolding of cerebral cortex, while CT reflects the density and distribution of neurons (Winkler et al., 2018). Joint analysis of CSA and CT may provide new insights into cortical changes associated with PD asymmetry.

Moreover, PD involves altered connections between various brain regions, it can also be considered a brain network disorder (Canu et al., 2015; Wang M. et al., 2016; Ji et al., 2018). The asymmetry in PD may result from the unequal degeneration of midbrain dopaminergic neurons, but it remains unknown how this localized structural damage leads to abnormalities in the entire brain network (Li et al., 2020). Structural covariance networks (SCNs) provide an effective means to explore the lateralization of PD from a network perspective by revealing coordinated morphological variations across brain regions (Vijayakumar et al., 2021). Studies have reported increased clustering coefficient and path length in SCNs of PD patients compared to healthy controls, suggesting network-level abnormalities associated with disease progression (Pereira et al., 2015; Zhang et al., 2015; Xu et al., 2017; Wu et al., 2018). Despite these findings, SCNs related to the lateralization of PD remain poorly understood.

Therefore, this study aims to analyze cortical structural changes in LPD and RPD patients using the SBM approach and to investigate differences in brain network topology between the two groups through SCNs analysis. We expect that these investigations will provide new insights into the mechanisms underlying the lateralization of PD.

## Materials and methods

### Participants

This study was approved by the local ethical committee of the First Affiliated Hospital of Zhengzhou University. In compliance with the Declaration of Helsinki, written informed consent was obtained from all subjects before participation. The inclusion criteria were as follows: (1) no significant cognitive impairment assessed by the Mini-Mental State Examination (MMSE); (2) right-handedness; and (3) no history of other psychiatric or neurological diseases. Subjects were excluded if they (1) had other diseases and treatments that could potentially affect brain function, such as atypical parkinsonism, cerebral trauma, stroke, and other diseases of the neurological system; (2) had contraindications to MRI or were unable to cooperate with an MRI scan and clinical assessments. All PD patients underwent assessment in a practically defined “off” state, achieved by withholding anti-parkinsonian medications for 12 h overnight (Espay et al., 2012), except during MRI acquisition. PD patients were divided into LPD and RPD groups based on the side of motor symptom onset. This classification was confirmed through retrospective chart reviews, patient self-reports, and early-stage clinical evaluations by experienced neurologists at our institution.

### MRI data acquisition and preprocessing

Anatomical 3D T1-weighted fast field echo (FFE) MRI images were acquired on a 3 T Siemens Verio scanner (Siemens, Erlangen, Germany) using a 32-channel receive coil in the Department of Medical Imaging, The First Affiliated Hospital of Zhengzhou University. A memory foam padding was used to minimize head motion, and earplugs were used to reduce scanner noise. The MRI parameters were as follows: 218 sagittal slices, repetition time (TR) = 1900 ms, echo time (TE) = 2.93 ms, thickness = 1.0 mm, no gap, flip angle = 9°, matrix size = 256 × 256 reconstructed to 352 × 352 over a 220-mm field of view, and voxel size = 0.625 × 0.625 × 1 mm^3^.

MRI data were preprocessed using FreeSurfer 7.4.1 to estimate CSA and CT (Dale et al., 1999; Fischl, 2012). FreeSurfer is open source software for accurate and automated human cortical thickness measurements and cross-subjects statistics based on cortical anatomy (Fischl and Dale, 2000). The suite offers both whole brain vertex-wise analysis, which localizes clusters across the whole cortical mantle and ROI-based analysis after automatically parcellating the cortex into regions based on standard anatomical and functional atlases. In short, image processing procedures included motion correction using the average of multiple volumetric images, skull and non-brain tissue stripping, automated Talairach transformation, subcortical white and deep grey matter segmentation, grey and white matter tessellation, automated topology correction, and surface deformation to optimize the grey/white and grey/cerebrospinal fluid boundaries. To ensure data quality, images were inspected for significant motion artifacts during preprocessing, and only those meeting quality standards were included for subsequent analysis. The quantitative measures of CSA and CT for cortical regions were defined using the Desikan atlas (Desikan et al., 2006).

### Constructing structural covariance networks

**Figure 1:**
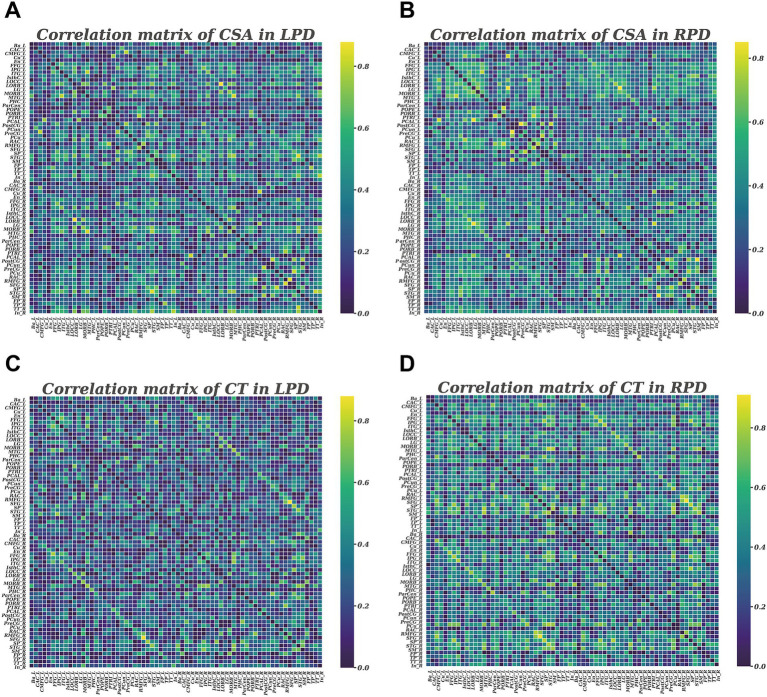
Correlation matrices with 68 × 68 for LPD and RPD groups based on cortical surface area **(A,B)** and cortical thickness **(C,D)**. These matrices display the Pearson correlation coefficients between pairs of regions in the network. The color bar represents the absolute value of the Pearson correlation coefficients, indicating the strength of the connections.

The Brain Connectivity Toolbox was employed to construct the SCNs based on CSA and CT (Rubinov and Sporns, 2010). For each group, a 68 × 68 correlation matrix was constructed by calculating Pearson correlation coefficients between CSA or CT values of each brain region. To emphasize the strength of structural covariance regardless of direction, the absolute values of these coefficients were taken, and the resulting matrix was then converted into a binary adjacency matrix by thresholding to values of 1 or 0 (Figure 1). Thresholds were defined as a network sparsity range from 0.1 to 0.4 (increments of 0.01), which ensured that LPD and RPD networks had the same number of nodes and edges at each density. The chosen sparsity range allows the small-world network architectures to be properly estimated, and the number of spurious edges in each network is minimized, as indicated in previous studies (Achard and Bullmore, 2007; He et al., 2007).

### Graph-based network analysis

As measures of network integration, we calculated the normalized characteristic path length, defined as the shortest path length between all pairs of nodes, and global efficiency, which measures how efficiently information is communicated between nodes. To assess network segregation, we analyzed the normalized clustering coefficient which evaluates the influence of different paths based on the connection weights of the node’s neighbors, and local efficiency, defined as the number of connections in the neighborhood of a certain node divided by the maximum number of possible connections between the neighbors of this node. To evaluate the extent of network modular organization, we computed modularity, which quantifies the strength of division of a network into distinct functional modules or communities. Small-worldness, reflecting the balance between network integration and segregation, was also computed. To explore group differences in nodal network measures, we examined nodal degree, nodal efficiency, and nodal betweenness centrality.

### Statistical analysis

The statistical analyses of demographic and clinical indices were conducted using the SPSS version 22.0 (SPSS Inc., Chicago, IL, United States). The normal distribution of the data was assessed by Shapiro–Wilk test. Group differences in age, years of education, age at onset, Unified Parkinson’s Disease Rating Scale motor section (UPDRS-III), Beck Depression Inventory (BDI), and levodopa equivalent daily dose (LEDD) were analyzed with unpaired two-tailed t-tests. The Mann–Whitney U test was used to analyze differences in disease duration, Parkinson’s Disease Questionnaire (PDQ-39), MMSE, and Hoehn and Yahr stage. A two-tailed *p* < 0.05 was considered statistically significant.

To assess group differences in CT and CSA, we conducted whole-brain vertex-wise analysis using the graphical user interface of FreeSurfer known as QDEC (Query, Design, Estimate, Contrast) (van Eijndhoven et al., 2013; Bruno et al., 2017). We used a general linear model (GLM) to compare CSA and CT between LPD and RPD groups with age and sex as covariates. The Monte Carlo Null-Z Simulation was employed to control for multiple comparisons (10,000 iterations, cluster-forming *p* < 0.05, cluster-wise corrected *p* < 0.05). Then, partial correlation analyses were conducted separately for the LPD and RPD groups to investigate associations between the CSA and CT of regions showing significant group differences and clinical variables (age of onset, duration, MDS-UPDRS III, PDQ-39, BDI, MMSE, and LEDD), with age and sex as covariates. A significance threshold of *p* < 0.05 was adopted for these exploratory analyses, without correction for multiple comparisons.

To assess the statistical significance of group differences in all network parameters, we used a non-parametric permutation test with 2,000 repetitions (He et al., 2008; Zhang et al., 2019). For each repetition, the corrected CSA and CT values of each subject were randomly reassigned to one of two new groups with the same number as the original LPD and RPD groups, and then the correlation matrices were recalculated for the two new groups. For the two new groups, network parameters were calculated and differences were compared at each sparsity. The area under the curve (AUC) was computed using the trapezoidal rule with a step size of 0.01 to integrate the group difference trajectories across all sparsity thresholds, summarizing cumulative differences over the entire density range (Zhang et al., 2019). The statistical threshold was set at *p* < 0.05 for group differences in global network parameters. For regional network parameters, a *p* < 0.05 significance level was applied following false discovery rate (FDR) correction using the Benjamini-Hochberg method.

## Results

### Demographic and clinical characteristics

There were 50 cases in LPD group (1 excluded from 51 recruited due to image quality issues) and 49 cases in RPD group. The demographic and clinical characteristics of participants are summarized in Table 1. Age, gender, disease duration, years of education, age at onset, MDS-UPDRS III score, PDQ-39 score, Hoehn and Yahr stage, MMSE score and BDI score were comparable between the two groups (*p* > 0.05; Table 1).

**Table 1:** Demographic and clinical data of study groups.

Characteristic	LPD (*N* = 50)	RPD (*N* = 49)	*p* (LPD vs. RPD)
Age, years, mean ± SD	64.10 ± 7.957	62.90 ± 10.574	0.524
Gender, F / M	23 / 27	24 / 25	–
Education, years, (IR)	6.00 (6.00–9.00)	9.00 (6.00–9.00)	0.522
Age of onset, years, mean ± SD	55.98 ± 8.498	54.98 ± 10.209	0.597
Duration, years, (IR)	7.00 (5.00–10.00)	7.00 (5.00–10.00)	0.682
UPDRS-III, mean ± SD	55.38 ± 12.227	53.35 ± 15.098	0.463
MMSE, (IR)	27.00 (22.00–28.00)	27.00 (24.00–28.00)	0.780
PDQ-39, (IR)	68.50 (51.75–88.25)	73 (50.50–91.50)	0.629
BDI, mean ± SD	19.16 ± 11.601	18.24 ± 9.148	0.664
LEDD, mg, mean ± SD	822.470 ± 361.0314	813.286 ± 341.9414	0.897
Hoehn and Yahr, (IR)	3.00 (2.50–4.00)	3.00 (2.50–4.00)	0.515

LPD, left-onset Parkinson’s disease; RPD, right-onset Parkinson’s disease; UPDRS-III, Unified Parkinson’s Disease Rating Scale motor section; MMSE, Mini-Mental State Examination; PDQ-39, Parkinson’s Disease Questionnaire; BDI, Beck Depression Inventory; LEDD, levodopa equivalent daily dose.

### Group differences in CSA and CT

**Figure 2:**
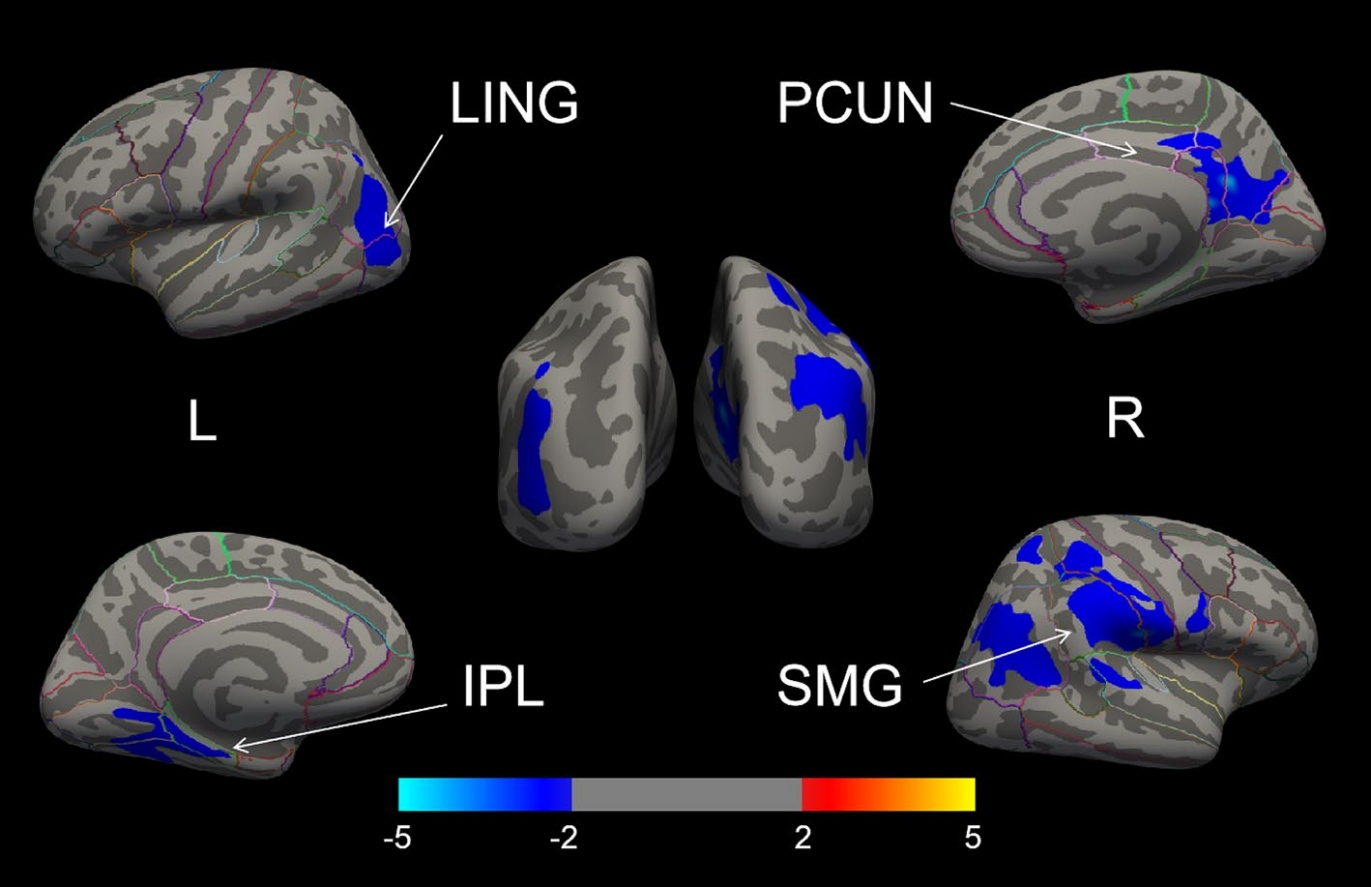
The brain regions with cortical surface area differences between the LPD and RPD groups (corrected using Monte Carlo Null-Z Simulation for *p* < 0.05). Blue (negative values) indicates a reduction in cortical surface area in the LPD compared to RPD group. SMG, supramarginal gyrus; PCUN, precuneus; IPL, inferior parietal lobule; LING, lingual gyrus.

The whole-brain vertex-wise analysis revealed that compared to RPD patients, the LPD patients exhibited 4 clusters with significantly smaller CSA as follows: cluster 1 in the right hemisphere was primarily located in the supramarginal gyrus (SMG); cluster 2 in the right hemisphere was located in the PCUN; cluster 3 in the left hemisphere was mainly in the inferior parietal lobule (IPL); and cluster 4 in the left hemisphere was in the lingual gyrus (LING). All clusters were corrected using Monte Carlo simulations at *p* < 0.05 (Figure 2, Table 2). However, the vertex-wise comparisons with correction for multiple comparisons of CT found no differences between the two groups.

**Table 2:** Significant clusters with altered cortical surface area in LPD versus RPD.

Brain regions	Maximum vertex coordinate of significant clusters			Size (mm^2^)	*P*-value for CWP
	MNIX	MNIY	MNIZ		
Cortical surface area					
LPD < RPD					
Right SMG	56.5	−19	17.3	9956.05	0.0001
Right PCUN	7.6	−54.5	18.9	2781.66	0.0105
Left LING	−32.1	−50.4	-6.7	2335.01	0.0301
Left IPL	−40.2	−69.3	17.5	2328.80	0.0303

All clusters survived correction for multiple comparisons using a Monte Carlo simulation, resulting in a corrected cluster-wise p < 0.05. CWP, cluster-wise probability; LPD, left-onset Parkinson’s disease; RPD, right-onset Parkinson’s disease; SMG, supramarginal gyrus; PCUN, precuneus; LING, lingual gyrus; IPL, inferior parietal lobule.

### Correlation between morphometrical alterations and clinical variables

**Figure 3:**
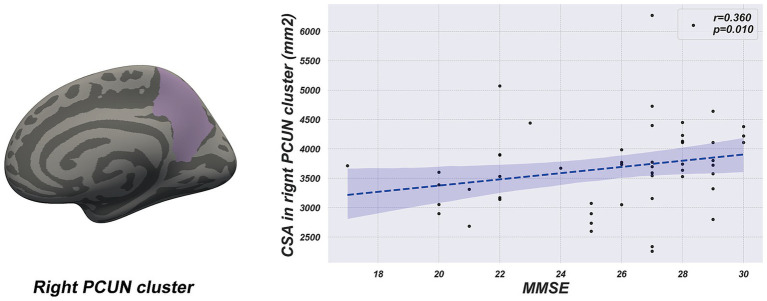
The scatter plot shows a positive correlation between cortical surface area of the right precuneus cluster and MMSE score in the LPD group.

Partial correlation analyses, adjusted for age and sex, were conducted separately for the LPD and RPD groups to examine relationships between the CSA of the four regions with significant group differences (right SMG, right PCUN, left IPL, and left LING) and clinical variables (age of onset, duration, MDS-UPDRS III, PDQ-39, BDI, MMSE, and LEDD). In the LPD group, the CSA of the right PCUN was significantly positively correlated with MMSE score (*r* = 0.360, *p* = 0.01) (Figure 3). No other significant correlations were observed between the CSA of these regions and any clinical variables in the LPD group (*p* > 0.05). In the RPD group, no significant correlations were found between the CSA of the four regions and any clinical variables (*p* > 0.05). A summary of all tested correlations for both groups is provided in Supplementary Table S1.

### Global network characteristics

**Figure 4:**
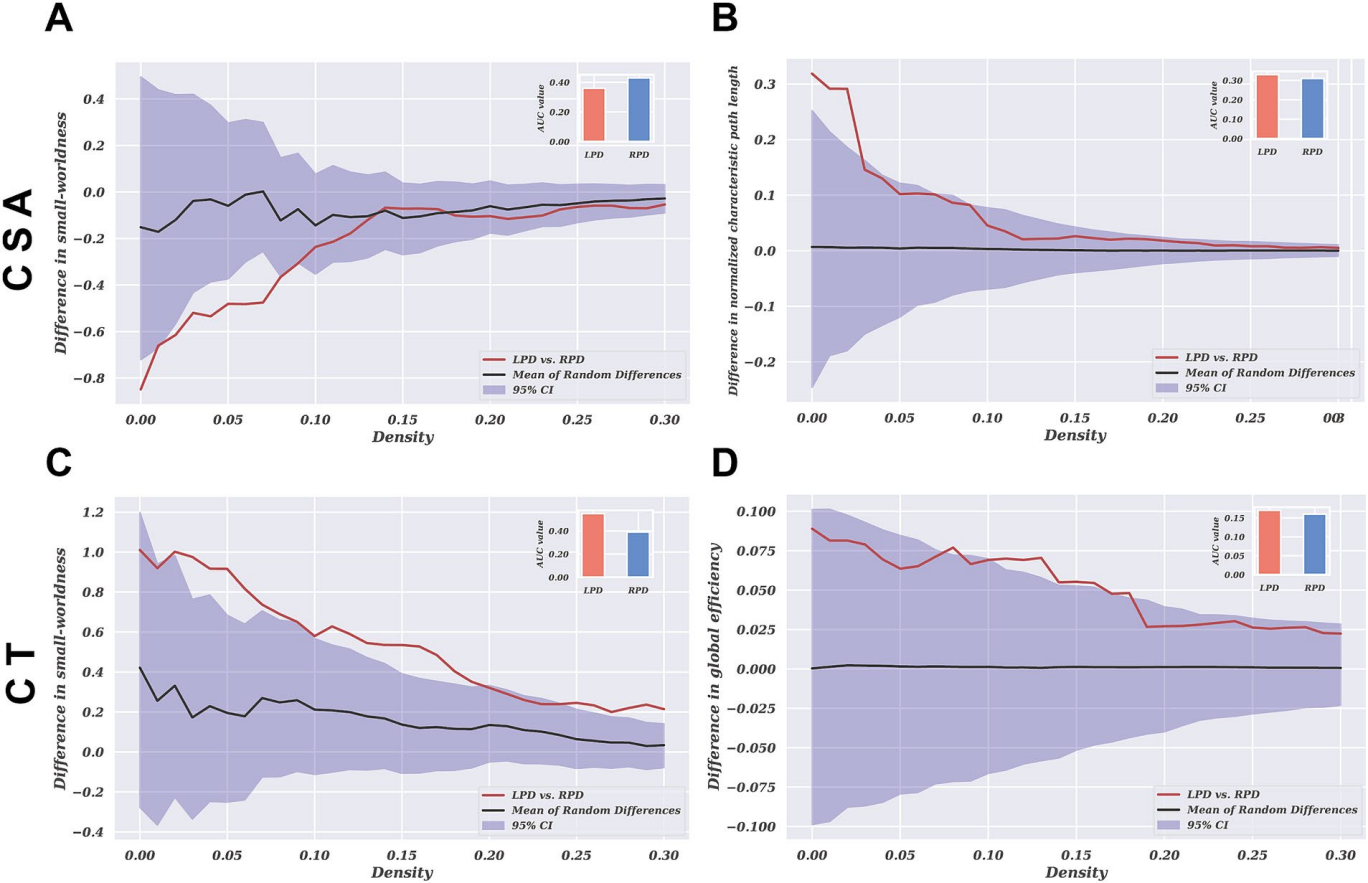
The group differences in network parameters of structural covariance networks based on CSA and CT within the range of 10–40% network sparsity include **(A)** and **(C)** small-worldness, **(B)** normalized characteristic path length, and **(D)** global efficiency. The upper and lower blue bands represent the 95% confidence intervals, while the middle black line indicates the mean difference after 2,000 permutations. The red line represents the actual group difference, and if it falls outside the confidence interval, it indicates that the group difference is significant at the current threshold (*p* < 0.05). Positive values indicate LPD > RPD, and negative values indicate LPD < RPD. The subplots show the group differences in the AUC values for each measure of the SCNs.

The global network parameter changes and between-group differences for CSA and CT in the LPD and RPD patients across a sparsity range of 0.10 to 0.40 are shown in Figure 4. Both groups exhibited small-world properties in their SCNs, with a small-world index greater >1. For CSA-based networks, AUC analysis revealed that the normalized characteristic path length was significantly increased in LPD patients compared to RPD (*p* = 0.024), while the small-world index was significantly higher in RPD patients (*p* = 0.037). Conversely, for CT-based networks, AUC analysis revealed that the small-world index and global efficiency were significantly higher in LPD patients compared to RPD (*p* = 0.006 and *p* = 0.032, respectively). For the remaining global network parameters, no significant between-group differences were observed between LPD and RPD patients (all *p* > 0.05; Supplementary Figures S1, S2).

### Regional network characteristics

We investigated the networks (sparsity = 0.01) for between-group differences in regional network measures, including nodal betweenness, nodal efficiency, and nodal degree. No significant differences in nodal characteristics were observed after correction for multiple comparisons in either CSA-based or CT-based networks between the two groups (*p* > 0.05; Supplementary Figure S3).

## Discussion

To the best of our knowledge, this study is the first to investigate cortical morphometric alterations in CSA associated with the lateralization of PD. Additionally, this study is the first to reveal abnormal topological organization of SCNs between LPD and RPD patients. The results showed that LPD patients exhibited significantly smaller CSA in the right PCUN, right SMG, left IPL, and left LING compared to RPD patients. In our SCNs analysis, LPD patients exhibited increased normalized characteristic path length and decreased small-world index in CSA-based networks, while in CT-based networks, they showed increased small-world index and global efficiency compared to RPD. No significant differences in nodal characteristics were observed in either CSA-based or CT-based networks between the two groups. These findings provide novel multiscale evidence for the mechanisms underlying symptom lateralization in PD.

### Analysis of specific regional morphological changes

Previous studies have indicated that the right PCUN is involved in visuospatial memory and attention allocation, and its atrophy has been linked to cognitive impairments in PD (Noh et al., 2014; Mak et al., 2015; Aracil-Bolaños et al., 2019). In our research, LPD patients exhibited a significant reduction in the CSA of the right precuneus compared to RPD patients. Moreover, partial correlation analysis showed a positive relationship between the CSA of the right precuneus and MMSE score in LPD patients, suggesting that atrophy in this region might contribute to cognitive impairment. Additionally, a study using a resting-state structural connectome, constructed by integrating diffusion tensor imaging tractography with resting-state data, reported decreased degree centrality in the right PCUN of LPD patients (Zhang et al., 2022). This reduction in connectivity, reflecting a blend of structural white matter pathways and functional correlations, supports our findings of structural changes in the same region. These results suggest that the right PCUN could be an important brain region for cognitive changes in LPD patients, offering new insights into the pathological mechanisms underlying cognitive impairments in PD.

Additionally, our study revealed that LPD patients exhibited reduced CSA in the right SMG, left IPL, and left LING compared to RPD patients. Structural changes in these regions may be associated with the occurrence of hallucinations in PD patients, a common non-motor symptom of the disease (Weil et al., 2016). Meta-analyses have demonstrated significant gray matter reductions in the right SMG and left LING in PD patients with hallucinations (Rollins et al., 2019). Similarly, Goldman et al. reported markedly decreased gray matter volume in the left IPL of PD patients with hallucinations compared to those without hallucinations (Goldman et al., 2014). Additionally, Stavitsky et al. found that LPD patients are more prone to hallucinations than RPD patients (Stavitsky et al., 2008). Previous studies have shown that CSA is strongly correlated with gray matter volume and can reflect the extent of atrophy in specific brain regions (Winkler et al., 2010). In our study, the brain regions where LPD patients exhibited significant reductions in CSA correspond to the areas of gray matter loss reported in the aforementioned studies. This finding suggests that CSA reductions in these regions may be closely related to the occurrence of hallucinations in LPD patients. However, due to the lack of clinical data related to visual hallucinations, we cannot further analyze the direct association between CSA changes in relevant brain regions and hallucinations.

### Alterations in global network parameters

There is increasing evidence suggesting that the pathophysiological mechanisms of PD are associated with abnormalities in cortical morphology and connectivity across widespread brain regions (Jankovic, 2008). SCNs analysis offers an effective means to explore PD from a network perspective by revealing co-variations in brain region morphology (Vijayakumar et al., 2021). In our SCNs analysis, we found that both LPD and RPD patients exhibit small-world topological properties in their SCNs. Small-world topology reflects an optimal balance between local segregation and global integration of structural covariation (Achard et al., 2006; Kaiser and Hilgetag, 2006). This finding is consistent with previous studies on SCNs in PD patients (Pereira et al., 2015; Zhang et al., 2015; Xu et al., 2017; Wu et al., 2018). However, the topological structures of CSA-based and CT-based networks showed significantly different patterns between LPD and RPD patients.

Our study revealed that in CSA-based networks, the normalized characteristic path length was significantly higher in LPD compared to RPD patients, while the small-world index was significantly higher in RPD than in LPD patients. The normalized characteristic path length reflects the compactness of covariance patterns across regions, with higher values indicating less coordinated structural covariation (Suo et al., 2021). This finding suggests that LPD patients exhibit more fragmented CSA covariation patterns, whereas RPD patients demonstrate better integration of structural covariance across cortical regions. Hall et al. found that PD patients with visual hallucinations showed altered structural covariance in vision-related networks (Hall et al., 2019). When contextualized with our observed CSA differences, these fragmented covariance patterns in LPD may reflect impaired neurodevelopmental coordination between key regions implicated in perceptual processing. This may imply that the fragmented covariance patterns observed in LPD patients are associated with the occurrence of hallucinations.

However, in CT-based networks, LPD patients exhibited significantly higher small-world indices and global efficiency compared to RPD patients. This suggests that CT-based networks in LPD patients display a more optimized topological organization, reflecting greater covariance integration across regions. This dissociation in network topology between CSA-based and CT-based networks reflects the distinct characteristics of these two morphological metrics. Previous studies have shown that CSA and CT are orthogonal components influenced by different genetic and biological processes, with independent patterns of change during aging and disease progression (Dickerson et al., 2009; Panizzon et al., 2009; Storsve et al., 2014). Similar dissociated morphological alterations have been observed in other diseases (Park et al., 2009; Abé et al., 2016). The findings of this study suggest that the changing trends in CSA and CT in PD may reflect distinct pathological processes, and their independently divergent nature deserves further investigation.

### Preservation of regional network architecture

Notably, although global network parameters revealed lateralization-related differences, there were no significant intergroup differences in regional network metrics such as nodal efficiency, modularity, and clustering coefficients. This suggests that despite altered global integration patterns, the fundamental community architecture of SCNs remains preserved between LPD and RPD patients. Modularity reflects the degree to which a network is compartmentalized into distinct functional subsystems, while the clustering coefficient quantifies local connectivity (Alexander-Bloch et al., 2013). The absence of differences in modularity or clustering coefficients implies that the lateralization of PD symptoms primarily affects the efficiency of information integration across distributed regions, rather than disrupting the organization of local communities. This observation is consistent with previous studies. For instance, Frigerio et al. (2024) found that, although there were significant differences in global network parameters such as characteristic path length and global efficiency between patients with PD and healthy controls, no significant differences were observed in regional network metrics. This suggests that the local network structure of patients with PD is largely preserved. These findings suggest that PD-related lateralization may primarily target the coordination of large-scale network integration while preserving local structural covariance patterns.

## Limitations

This study has several limitations that need to be addressed. Firstly, the relatively small sample size may limit the generalizability of the results, and future studies with larger, earlier-stage cohorts are necessary to clarify how motor symptom laterality influences brain structure over time, distinct from overall disease progression. Secondly, SCNs analysis can characterize brain structure but fail to capture dynamic network changes. Therefore, integrating resting-state fMRI could address this limitation. Thirdly, the MMSE is not sensitive enough to assess specific cognitive domains, and thus future studies should include more detailed neuropsychological assessments. Finally, the calculation of network parameters relies on small-sample group-level data, limiting individual-level analysis of clinical-network relationships.

## Conclusion

This study employed SBM and SCNs to investigate differences in cortical structural characteristics and brain network topological properties between LPD and RPD patients. The results revealed that LPD patients exhibited significant reductions in CSA in the right PCUN, right SMG, left IPL, and left LING, which may be linked to cognitive impairments and hallucinations among non-motor symptoms of PD. Moreover, divergent global network properties in CSA-based and CT-based networks suggest PD lateralization may influence the global organization of covariance patterns more than the local segregation into distinct communities. These findings offer new insights into the mechanisms of symptom lateralization in PD from the perspective of brain regional structure and network topology.

## Data Availability

The original contributions presented in the study are included in the article/Supplementary material, further inquiries can be directed to the corresponding author.

## Supplementary material

SUPPLEMENTARY FIGURE S1 The group differences in network parameters of structural covariance networks based on CSA within the range of 10%-40% network sparsity include **(A)** clustering coefficient, **(B)** global efficiency, **(C)** local efficiency, **(D)** normalized clustering coefficient, **(E)** characteristic path length and **(F)** modularity. The upper and lower blue bands represent the 95% confidence intervals, while the middle black line indicates the mean difference after 2,000 permutations. The red line represents the actual difference between groups, and if it falls within the confidence interval, it indicates that the group difference is not significant at the current threshold (*p* > 0.05). Positive values indicate LPD > RPD, and negative values indicate LPD < RPD. The subplots show the group differences in the AUC values for each measure of the SCNs.

SUPPLEMENTARY FIGURE S2 The group differences in network parameters of structural covariance networks based on CT within the range of 10%-40% network sparsity include **(A)** clustering coefficient, **(B)** local efficiency, **(C)** normalized clustering coefficient, **(D)** normalized characteristic path length, **(E)** characteristic path length and **(F)** modularity. The upper and lower blue bands represent the 95% confidence intervals, while the middle black line indicates the mean difference after 2,000 permutations. The red line represents the actual difference between groups, and if it falls within the confidence interval, it indicates that the group difference is not significant at the current threshold (*p* > 0.05). Positive values indicate LPD > RPD, and negative values indicate LPD < RPD. The subplots show the group differences in the AUC values for each measure of the SCNs.

SUPPLEMENTARY FIGURE S3 Nodal network measures of CSA and CT based networks in the LPD and RPD groups. Each circle represents a brain region, with a total of 68 regions analyzed. The figure displays the distribution of nodal degree, betweenness centrality, and nodal efficiency, accompanied by *p*-values derived from permutation tests (all *p* > 0.05 after FDR correction). These *p*-values are mapped onto brain regions, with colors indicating the *p*-value range (blue: *p* = 0.1, red: *p* = 1).

## References

[ref1] Aarsland D. Batzu L. Halliday G. M. Geurtsen G. J. Ballard C. Ray Chaudhuri K.. (2021). Parkinson disease-associated cognitive impairment. Nat. Rev. Dis. Primers 7:47. doi: 10.1038/s41572-021-00280-3, PMID: 34210995

[ref2] Abé C. Ekman C. J. Sellgren C. Petrovic P. Ingvar M. Landén M. (2016). Cortical thickness, volume and surface area in patients with bipolar disorder types I and II. J. Psychiatry Neurosci. 41, 240–250. doi: 10.1503/jpn.150093, PMID: 26645741 PMC4915933

[ref3] Achard S. Bullmore E. (2007). Efficiency and cost of economical brain functional networks. PLoS Comput. Biol. 3:e17. doi: 10.1371/journal.pcbi.0030017, PMID: 17274684 PMC1794324

[ref4] Achard S. Salvador R. Whitcher B. Suckling J. Bullmore E. (2006). A resilient, low-frequency, small-world human brain functional network with highly connected association cortical hubs. J. Neurosci. 26, 63–72. doi: 10.1523/jneurosci.3874-05.2006, PMID: 16399673 PMC6674299

[ref5] Alexander-Bloch A. Giedd J. N. Bullmore E. (2013). Imaging structural co-variance between human brain regions. Nat. Rev. Neurosci. 14, 322–336. doi: 10.1038/nrn3465, PMID: 23531697 PMC4043276

[ref6] Amick M. M. Grace J. Chou K. L. (2006). Body side of motor symptom onset in Parkinson's disease is associated with memory performance. J. Int. Neuropsychol. Soc. 12, 736–740. doi: 10.1017/s1355617706060875, PMID: 16961953

[ref7] Aracil-Bolaños I. Sampedro F. Marín-Lahoz J. Horta-Barba A. Martínez-Horta S. Botí M.. (2019). A divergent breakdown of neurocognitive networks in Parkinson's disease mild cognitive impairment. Hum. Brain Mapp. 40, 3233–3242. doi: 10.1002/hbm.24593, PMID: 30938027 PMC6865605

[ref8] Baumann C. R. Held U. Valko P. O. Wienecke M. Waldvogel D. (2014). Body side and predominant motor features at the onset of Parkinson's disease are linked to motor and nonmotor progression. Mov. Disord. 29, 207–213. doi: 10.1002/mds.25650, PMID: 24105646

[ref9] Bruno J. L. Hosseini S. M. H. Saggar M. Quintin E. M. Raman M. M. Reiss A. L. (2017). Altered brain network segregation in fragile X syndrome revealed by structural Connectomics. Cereb. Cortex 27, bhw055–bhw2259. doi: 10.1093/cercor/bhw055, PMID: 27009247 PMC5963822

[ref10] Canu E. Agosta F. Sarasso E. Volontè M. A. Basaia S. Stojkovic T.. (2015). Brain structural and functional connectivity in Parkinson's disease with freezing of gait. Hum. Brain Mapp. 36, 5064–5078. doi: 10.1002/hbm.22994, PMID: 26359798 PMC6869160

[ref11] Dale A. M. Fischl B. Sereno M. I. (1999). Cortical surface-based analysis. I. Segmentation and surface reconstruction. Neuroimage 9, 179–194. doi: 10.1006/nimg.1998.0395, PMID: 9931268

[ref12] Desikan R. S. Ségonne F. Fischl B. Quinn B. T. Dickerson B. C. Blacker D.. (2006). An automated labeling system for subdividing the human cerebral cortex on MRI scans into gyral based regions of interest. NeuroImage 31, 968–980. doi: 10.1016/j.neuroimage.2006.01.021, PMID: 16530430

[ref13] Dickerson B. C. Feczko E. Augustinack J. C. Pacheco J. Morris J. C. Fischl B.. (2009). Differential effects of aging and Alzheimer's disease on medial temporal lobe cortical thickness and surface area. Neurobiol. Aging 30, 432–440. doi: 10.1016/j.neurobiolaging.2007.07.022, PMID: 17869384 PMC3703585

[ref14] Djaldetti R. Ziv I. Melamed E. (2006). The mystery of motor asymmetry in Parkinson's disease. Lancet Neurol. 5, 796–802. doi: 10.1016/s1474-4422(06)70549-x, PMID: 16914408

[ref15] Espay A. J. Fasano A. van Nuenen B. F. Payne M. M. Snijders A. H. Bloem B. R. (2012). "On" state freezing of gait in Parkinson disease: a paradoxical levodopa-induced complication. Neurology 78, 454–457. doi: 10.1212/WNL.0b013e3182477ec0, PMID: 22262741 PMC3466608

[ref16] Fischl B. (2012). FreeSurfer. NeuroImage 62, 774–781. doi: 10.1016/j.neuroimage.2012.01.021, PMID: 22248573 PMC3685476

[ref17] Fischl B. Dale A. M. (2000). Measuring the thickness of the human cerebral cortex from magnetic resonance images. Proc. Natl. Acad. Sci. USA 97, 11050–11055. doi: 10.1073/pnas.200033797, PMID: 10984517 PMC27146

[ref18] Frigerio I. Broeders T. A. A. Lin C. P. Bouwman M. M. A. Koubiyr I. Barkhof F.. (2024). Pathologic substrates of structural brain network resilience and topology in Parkinson disease decedents. Neurology 103:e209678. doi: 10.1212/wnl.0000000000209678, PMID: 39042844 PMC11314958

[ref19] Goldman J. G. Stebbins G. T. Dinh V. Bernard B. Merkitch D. deToledo-Morrell L.. (2014). Visuoperceptive region atrophy independent of cognitive status in patients with Parkinson's disease with hallucinations. Brain 137, 849–859. doi: 10.1093/brain/awt360, PMID: 24480486 PMC3983409

[ref20] Goto M. Abe O. Hagiwara A. Fujita S. Kamagata K. Hori M.. (2022). Advantages of using both voxel- and surface-based morphometry in cortical morphology analysis: a review of various applications. Magn. Reson. Med. Sci. 21, 41–57. doi: 10.2463/mrms.rev.2021-0096, PMID: 35185061 PMC9199978

[ref21] Hall J. M. O'Callaghan C. Muller A. J. Ehgoetz Martens K. A. Phillips J. R. Moustafa A. A.. (2019). Changes in structural network topology correlate with severity of hallucinatory behavior in Parkinson's disease. Netw. Neurosci. 3, 521–538. doi: 10.1162/netn_a_00078, PMID: 30984905 PMC6444885

[ref22] Harris E. McNamara P. Durso R. (2013). Apathy in patients with Parkinson disease as a function of side of onset. J. Geriatr. Psychiatry Neurol. 26, 95–104. doi: 10.1177/0891988713481267, PMID: 23584852

[ref23] He Y. Chen Z. J. Evans A. C. (2007). Small-world anatomical networks in the human brain revealed by cortical thickness from MRI. Cereb. Cortex 17, 2407–2419. doi: 10.1093/cercor/bhl149, PMID: 17204824

[ref24] He Y. Chen Z. Evans A. (2008). Structural insights into aberrant topological patterns of large-scale cortical networks in Alzheimer's disease. J. Neurosci. 28, 4756–4766. doi: 10.1523/jneurosci.0141-08.2008, PMID: 18448652 PMC6670444

[ref25] Jankovic J. (2008). Parkinson's disease: clinical features and diagnosis. J. Neurol. Neurosurg. Psychiatry 79, 368–376. doi: 10.1136/jnnp.2007.131045 18344392

[ref26] Ji G. J. Hu P. Liu T. T. Li Y. Chen X. Zhu C.. (2018). Functional connectivity of the Corticobasal ganglia-Thalamocortical network in Parkinson disease: a systematic review and Meta-analysis with cross-validation. Radiology 287, 973–982. doi: 10.1148/radiol.2018172183, PMID: 29514016

[ref27] Kaiser M. Hilgetag C. C. (2006). Nonoptimal component placement, but short processing paths, due to long-distance projections in neural systems. PLoS Comput. Biol. 2:e95. doi: 10.1371/journal.pcbi.0020095, PMID: 16848638 PMC1513269

[ref28] Kim J. S. Yang J. J. Lee J. M. Youn J. Kim J. M. Cho J. W. (2014). Topographic pattern of cortical thinning with consideration of motor laterality in Parkinson disease. Parkinsonism Relat. Disord. 20, 1186–1190. doi: 10.1016/j.parkreldis.2014.08.021, PMID: 25231669

[ref29] Lee E. Y. Sen S. Eslinger P. J. Wagner D. Kong L. Lewis M. M.. (2015). Side of motor onset is associated with hemisphere-specific memory decline and lateralized gray matter loss in Parkinson's disease. Parkinsonism Relat. Disord. 21, 465–470. doi: 10.1016/j.parkreldis.2015.02.008, PMID: 25749355 PMC4424064

[ref30] Li K. Su W. Chen M. Li C. M. Ma X. X. Wang R.. (2020). Abnormal spontaneous brain activity in left-onset Parkinson disease: a resting-state functional MRI study. Front. Neurol. 11:727. doi: 10.3389/fneur.2020.00727, PMID: 32849201 PMC7399038

[ref31] Mak E. Su L. Williams G. B. Firbank M. J. Lawson R. A. Yarnall A. J.. (2015). Baseline and longitudinal grey matter changes in newly diagnosed Parkinson's disease: ICICLE-PD study. Brain 138, 2974–2986. doi: 10.1093/brain/awv211, PMID: 26173861 PMC4671477

[ref32] Miki Y. Tsushima E. Foti S. C. Strand K. M. Asi Y. T. Yamamoto A. K.. (2021). Identification of multiple system atrophy mimicking Parkinson's disease or progressive supranuclear palsy. Brain 144, 1138–1151. doi: 10.1093/brain/awab017, PMID: 33822892 PMC8310424

[ref33] Noh S. W. Han Y. H. Mun C. W. Chung E. J. Kim E. G. Ji K. H.. (2014). Analysis among cognitive profiles and gray matter volume in newly diagnosed Parkinson's disease with mild cognitive impairment. J. Neurol. Sci. 347, 210–213. doi: 10.1016/j.jns.2014.09.049, PMID: 25451006

[ref34] Panizzon M. S. Fennema-Notestine C. Eyler L. T. Jernigan T. L. Prom-Wormley E. Neale M.. (2009). Distinct genetic influences on cortical surface area and cortical thickness. Cereb. Cortex 19, 2728–2735. doi: 10.1093/cercor/bhp026, PMID: 19299253 PMC2758684

[ref35] Park H. J. Lee J. D. Kim E. Y. Park B. Oh M. K. Lee S.. (2009). Morphological alterations in the congenital blind based on the analysis of cortical thickness and surface area. NeuroImage 47, 98–106. doi: 10.1016/j.neuroimage.2009.03.076, PMID: 19361567

[ref36] Pereira J. B. Aarsland D. Ginestet C. E. Lebedev A. V. Wahlund L. O. Simmons A.. (2015). Aberrant cerebral network topology and mild cognitive impairment in early Parkinson's disease. Hum. Brain Mapp. 36, 2980–2995. doi: 10.1002/hbm.22822, PMID: 25950288 PMC6869566

[ref37] Phillipps C. Longato N. Béreau M. Carrière N. Lagha-Boukbiza O. Mengin A. C.. (2020). Is motor side onset of Parkinson's disease a risk factor for developing impulsive-compulsive behavior?. A cross-sectional study. Mov. Disord. 35, 1080–1081. doi: 10.1002/mds.28053, PMID: 32311121

[ref38] Postuma R. B. Berg D. Stern M. Poewe W. Olanow C. W. Oertel W.. (2015). MDS clinical diagnostic criteria for Parkinson's disease. Mov. Disord. 30, 1591–1601. doi: 10.1002/mds.26424, PMID: 26474316

[ref39] Rollins C. P. E. Garrison J. R. Simons J. S. Rowe J. B. O'Callaghan C. Murray G. K.. (2019). Meta-analytic evidence for the plurality of mechanisms in transdiagnostic structural MRI studies of hallucination status. EClinicalMedicine 8, 57–71. doi: 10.1016/j.eclinm.2019.01.012, PMID: 31193632 PMC6537703

[ref40] Rubinov M. Sporns O. (2010). Complex network measures of brain connectivity: uses and interpretations. NeuroImage 52, 1059–1069. doi: 10.1016/j.neuroimage.2009.10.003, PMID: 19819337

[ref41] Stavitsky K. McNamara P. Durso R. Harris E. Auerbach S. Cronin-Golomb A. (2008). Hallucinations, dreaming, and frequent dozing in Parkinson disease: impact of right-hemisphere neural networks. Cogn. Behav. Neurol. 21, 143–149. doi: 10.1097/WNN.0b013e318185e698, PMID: 18797256 PMC2630478

[ref42] Storsve A. B. Fjell A. M. Tamnes C. K. Westlye L. T. Overbye K. Aasland H. W.. (2014). Differential longitudinal changes in cortical thickness, surface area and volume across the adult life span: regions of accelerating and decelerating change. J. Neurosci. 34, 8488–8498. doi: 10.1523/jneurosci.0391-14.2014, PMID: 24948804 PMC6608217

[ref43] Suo X. Lei D. Li N. Li W. Kemp G. J. Sweeney J. A.. (2021). Disrupted morphological grey matter networks in early-stage Parkinson's disease. Brain Struct. Funct. 226, 1389–1403. doi: 10.1007/s00429-020-02200-9, PMID: 33825053 PMC8096749

[ref44] van Eijndhoven P. van Wingen G. Katzenbauer M. Groen W. Tepest R. Fernández G.. (2013). Paralimbic cortical thickness in first-episode depression: evidence for trait-related differences in mood regulation. Am. J. Psychiatry 170, 1477–1486. doi: 10.1176/appi.ajp.2013.12121504, PMID: 23929204

[ref45] Verreyt N. Nys G. M. Santens P. Vingerhoets G. (2011). Cognitive differences between patients with left-sided and right-sided Parkinson's disease. A review. Neuropsychol Rev 21, 405–424. doi: 10.1007/s11065-011-9182-x, PMID: 21956794

[ref46] Vijayakumar N. Ball G. Seal M. L. Mundy L. Whittle S. Silk T. (2021). The development of structural covariance networks during the transition from childhood to adolescence. Sci. Rep. 11:9451. doi: 10.1038/s41598-021-88918-w, PMID: 33947919 PMC8097025

[ref47] Wang M. Jiang S. Yuan Y. Zhang L. Ding J. Wang J.. (2016). Alterations of functional and structural connectivity of freezing of gait in Parkinson's disease. J. Neurol. 263, 1583–1592. doi: 10.1007/s00415-016-8174-4, PMID: 27230857

[ref48] Wang Z. Luo X. G. Gao C. (2016). Utility of susceptibility-weighted imaging in Parkinson's disease and atypical parkinsonian disorders. Transl. Neurodegener. 5:17. doi: 10.1186/s40035-016-0064-2, PMID: 27761236 PMC5054585

[ref49] Wang J. Yang Q. X. Sun X. Vesek J. Mosher Z. Vasavada M.. (2015). MRI evaluation of asymmetry of nigrostriatal damage in the early stage of early-onset Parkinson's disease. Parkinsonism Relat. Disord. 21, 590–596. doi: 10.1016/j.parkreldis.2015.03.012, PMID: 25825242

[ref50] Weil R. S. Schrag A. E. Warren J. D. Crutch S. J. Lees A. J. Morris H. R. (2016). Visual dysfunction in Parkinson's disease. Brain 139, 2827–2843. doi: 10.1093/brain/aww175, PMID: 27412389 PMC5091042

[ref51] Winkler A. M. Greve D. N. Bjuland K. J. Nichols T. E. Sabuncu M. R. Håberg A. K.. (2018). Joint analysis of cortical area and thickness as a replacement for the analysis of the volume of the cerebral cortex. Cereb. Cortex 28, 738–749. doi: 10.1093/cercor/bhx308, PMID: 29190325 PMC5972607

[ref52] Winkler A. M. Kochunov P. Blangero J. Almasy L. Zilles K. Fox P. T.. (2010). Cortical thickness or grey matter volume? The importance of selecting the phenotype for imaging genetics studies. NeuroImage 53, 1135–1146. doi: 10.1016/j.neuroimage.2009.12.028, PMID: 20006715 PMC2891595

[ref53] Wu Q. Gao Y. Liu A. S. Xie L. Z. Qian L. Yang X. G. (2018). Large-scale cortical volume correlation networks reveal disrupted small world patterns in Parkinson's disease. Neurosci. Lett. 662, 374–380. doi: 10.1016/j.neulet.2017.10.032, PMID: 29061395

[ref54] Xu J. Zhang J. Zhang J. Wang Y. Zhang Y. Wang J.. (2017). Abnormalities in structural covariance of cortical Gyrification in Parkinson's disease. Front. Neuroanat. 11:12. doi: 10.3389/fnana.2017.00012, PMID: 28326021 PMC5339339

[ref55] Zhang X. Li R. Xia Y. Zhao H. Cai L. Sha J.. (2022). Topological patterns of motor networks in Parkinson's disease with different sides of onset: a resting-state-informed structural connectome study. Front. Aging Neurosci. 14:1041744. doi: 10.3389/fnagi.2022.1041744, PMID: 36389065 PMC9643776

[ref56] Zhang Y. Qiu T. Yuan X. Zhang J. Wang Y. Zhang N.. (2019). Abnormal topological organization of structural covariance networks in amyotrophic lateral sclerosis. Neuroimage Clin. 21:101619. doi: 10.1016/j.nicl.2018.101619, PMID: 30528369 PMC6411656

[ref57] Zhang L. Shen Q. Liao H. Li J. Wang T. Zi Y.. (2021). Aberrant changes in cortical complexity in right-onset versus left-onset Parkinson's disease in early-stage. Front. Aging Neurosci. 13:749606. doi: 10.3389/fnagi.2021.749606, PMID: 34819848 PMC8606890

[ref58] Zhang D. Wang J. Liu X. Chen J. Liu B. (2015). Aberrant brain network efficiency in Parkinson's disease patients with tremor: a multi-modality study. Front. Aging Neurosci. 7:169. doi: 10.3389/fnagi.2015.00169, PMID: 26379547 PMC4553412

