# Supplementary figures and images for: Changes of brain structure and structural covariance networks in Parkinson’s disease with different sides of onset
Source: Front Aging Neurosci. 2025 Apr 15;17:1564754. doi: 10.3389/fnagi.2025.1564754 (PMC12037599; doi:10.3389/fnagi.2025.1564754)

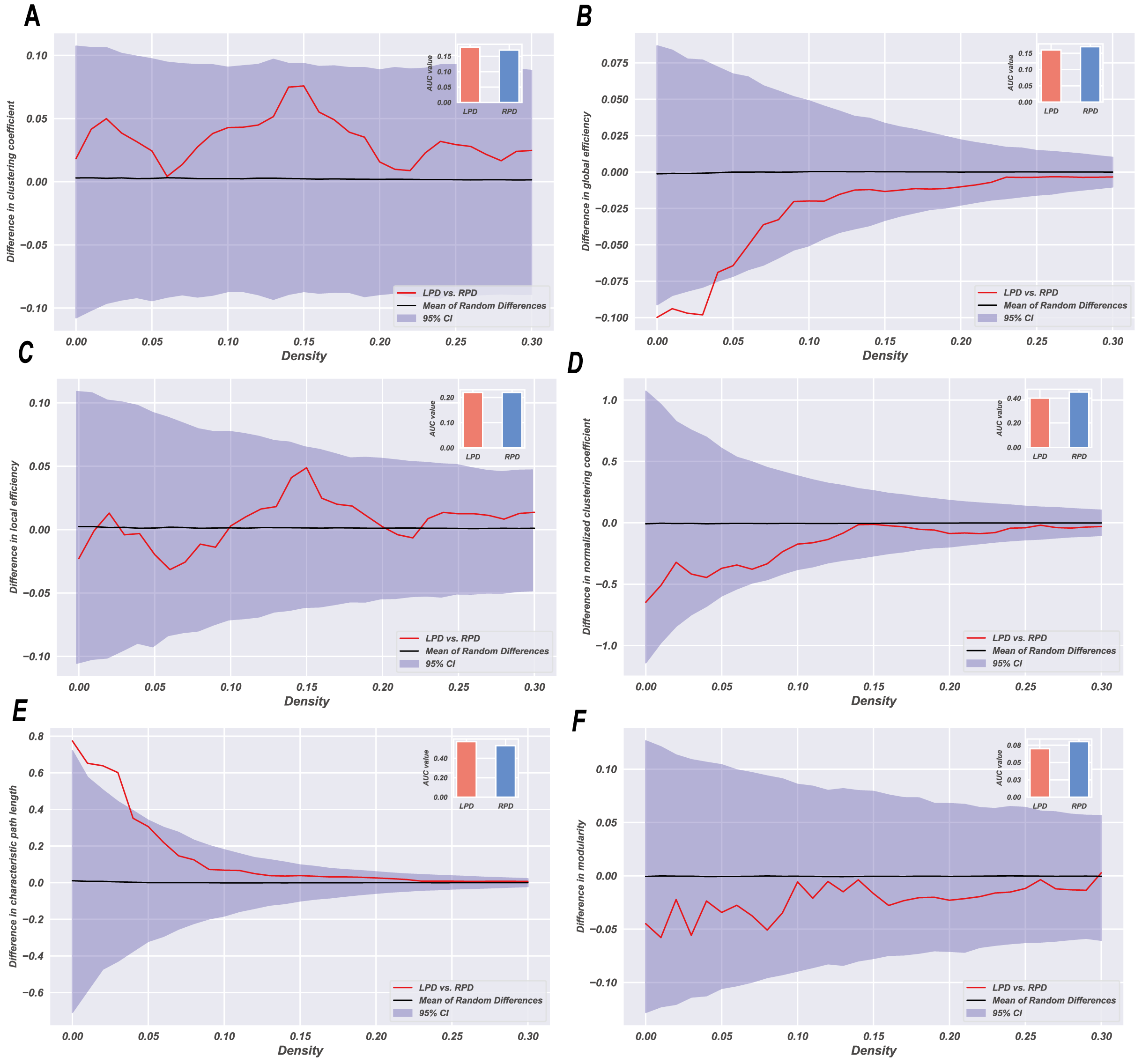

Supplement: SUPPLEMENTARY FIGURE S1 — The group differences in network parameters of structural covariance networks based on CSA within the range of 10%-40% network sparsity include (A) clustering coefficient, (B) global efficiency, (C) local efficiency, (D) normalized clustering coefficient, (E) characteristic path length and (F) modularity. The upper and lower blue bands represent the 95% confidence intervals, while the middle black line indicates the mean difference after 2,000 permutations. The red line represents the actual difference between groups, and if it falls within the confidence interval, it indicates that the group difference is not significant at the current threshold (p > 0.05). Positive values indicate LPD > RPD, and negative values indicate LPD < RPD. The subplots show the group differences in the AUC values for each measure of the SCNs. [file Image_1.tif]

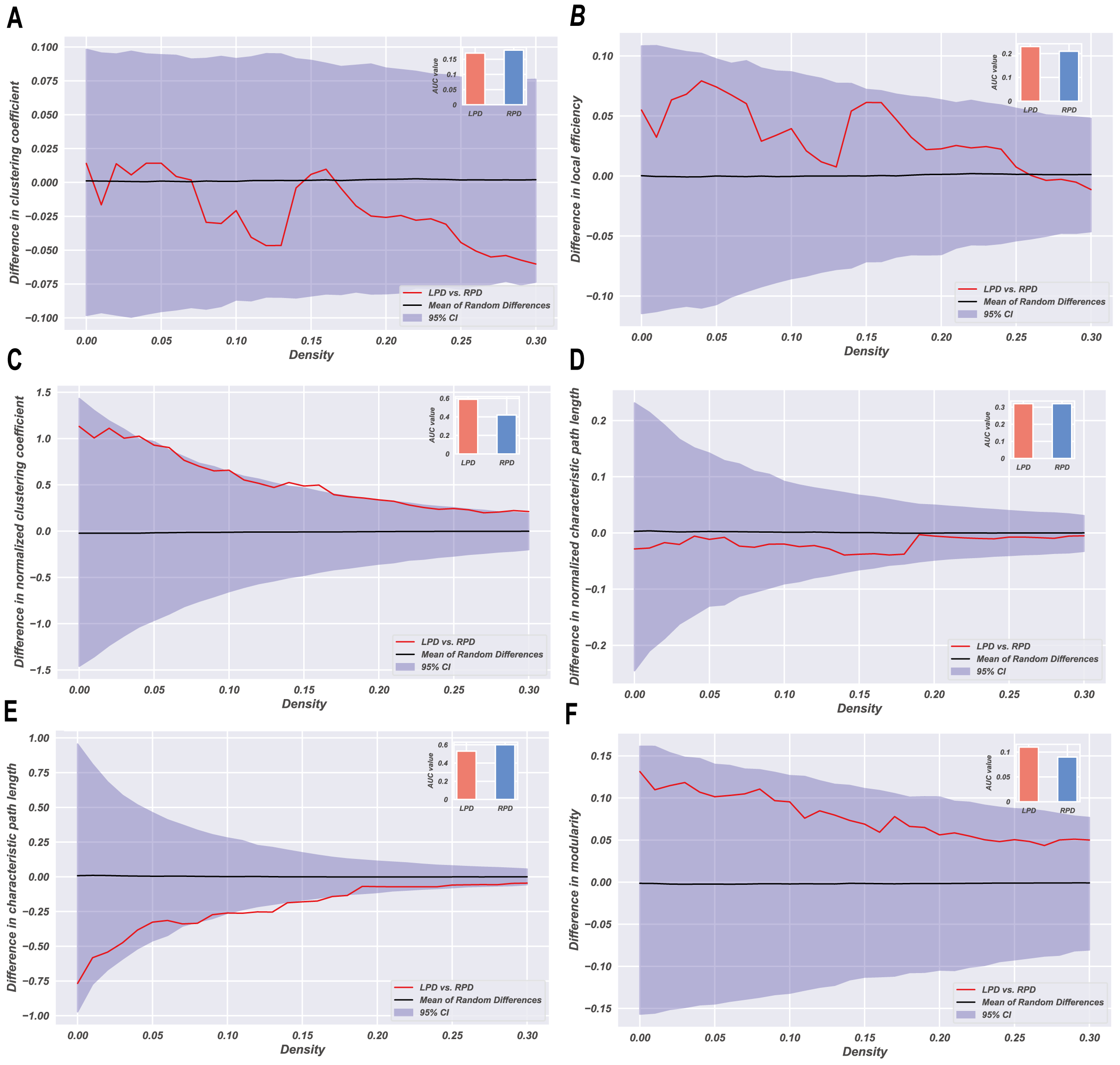

Supplement: SUPPLEMENTARY FIGURE S2 — The group differences in network parameters of structural covariance networks based on CT within the range of 10%-40% network sparsity include (A) clustering coefficient, (B) local efficiency, (C) normalized clustering coefficient, (D) normalized characteristic path length, (E) characteristic path length and (F) modularity. The upper and lower blue bands represent the 95% confidence intervals, while the middle black line indicates the mean difference after 2,000 permutations. The red line represents the actual difference between groups, and if it falls within the confidence interval, it indicates that the group difference is not significant at the current threshold (p > 0.05). Positive values indicate LPD > RPD, and negative values indicate LPD < RPD. The subplots show the group differences in the AUC values for each measure of the SCNs. [file Image_2.tif]

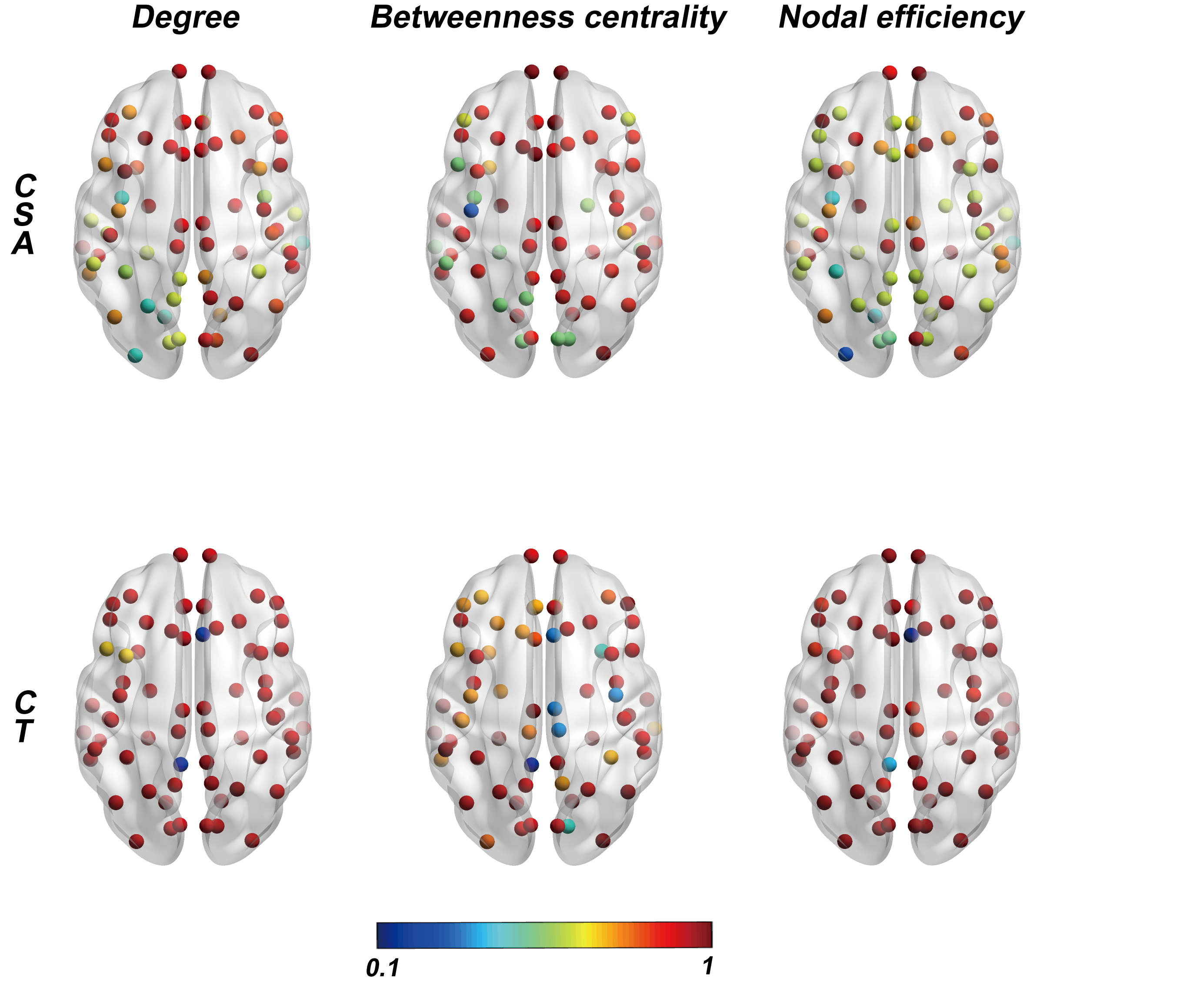

Supplement: SUPPLEMENTARY FIGURE S3 — Nodal network measures of CSA and CT based networks in the LPD and RPD groups. Each circle represents a brain region, with a total of 68 regions analyzed. The figure displays the distribution of nodal degree, betweenness centrality, and nodal efficiency, accompanied by p-values derived from permutation tests (all p > 0.05 after FDR correction). These p-values are mapped onto brain regions, with colors indicating the p-value range (blue: p = 0.1, red: p = 1). [file Image_3.tif]
