# Supplementary material for: Changes of brain structure and structural covariance networks in Parkinson’s disease with different sides of onset
Source: Front Aging Neurosci. 2025 Apr 15;17:1564754. doi: 10.3389/fnagi.2025.1564754 (PMC12037599; doi:10.3389/fnagi.2025.1564754)
Supplement: Supplementary file 4 [file Data_Sheet_1.docx]

Table S1. Partial correlation results in the LPD and RPD groups.

| Group | Brain regions | Clinical Variable | r | P-value (uncorrected) |
| --- | --- | --- | --- | --- |
| RPD | Left IPL | Age of onset | -0.265 | 0.066 |
|  |  | Duration | -0.104 | 0.475 |
|  |  | MDS-UPDRS III | 0.099 | 0.501 |
|  |  | PDQ-39 | 0.156 | 0.284 |
|  |  | BDI | -0.069 | 0.639 |
|  |  | MMSE | 0.085 | 0.560 |
|  |  | LEDD | -0.197 | 0.174 |
|  | Left LING | Age of onset | -0.090 | 0.537 |
|  |  | Duration | -0.199 | 0.171 |
|  |  | MDS-UPDRS III | -0.133 | 0.361 |
|  |  | PDQ-39 | 0.084 | 0.567 |
|  |  | BDI | -0.006 | 0.969 |
|  |  | MMSE | 0.269 | 0.063 |
|  |  | LEDD | 0.052 | 0.721 |
|  | Right PCUN | Age of onset | -0.269 | 0.061 |
|  |  | Duration | -0.228 | 0.115 |
|  |  | MDS-UPDRS III | -0.019 | 0.894 |
|  |  | PDQ-39 | 0.063 | 0.668 |
|  |  | BDI | 0.075 | 0.607 |
|  |  | MMSE | 0.266 | 0.065 |
|  |  | LEDD | -0.142 | 0.330 |
|  | Right SMG | Age of onset | -0.116 | 0.427 |
|  |  | Duration | -0.144 | 0.322 |
|  |  | MDS-UPDRS III | 0.026 | 0.862 |
|  |  | PDQ-39 | 0.161 | 0.270 |
|  |  | BDI | -0.041 | 0.780 |
|  |  | MMSE | 0.142 | 0.330 |
|  |  | LEDD | 0.034 | 0.817 |
| LPD | Left IPL | Age of onset | 0.045 | 0.754 |
|  |  | Duration | 0.016 | 0.914 |
|  |  | MDS-UPDRS III | -0.081 | 0.577 |
|  |  | PDQ-39 | 0.083 | 0.566 |
|  |  | BDI | -0.004 | 0.976 |
|  |  | MMSE | 0.212 | 0.139 |
|  |  | LEDD | 0.196 | 0.173 |
|  | Left LING | Age of onset | 0.100 | 0.490 |
|  |  | Duration | -0.025 | 0.861 |
|  |  | MDS-UPDRS III | -0.065 | 0.653 |
|  |  | PDQ-39 | 0.037 | 0.800 |
|  |  | BDI | -0.095 | 0.511 |
|  |  | MMSE | -0.107 | 0.458 |
|  |  | LEDD | 0.237 | 0.098 |
|  | Right PCUN | Age of onset | 0.057 | 0.694 |
|  |  | Duration | 0.049 | 0.736 |
|  |  | MDS-UPDRS III | -0.035 | 0.809 |
|  |  | PDQ-39 | 0.163 | 0.257 |
|  |  | BDI | -0.147 | 0.307 |
|  |  | MMSE | 0.360 | 0.010* |
|  |  | LEDD | 0.138 | 0.339 |
|  | Right SMG | Age of onset | 0.171 | 0.234 |
|  |  | Duration | 0.065 | 0.654 |
|  |  | MDS-UPDRS III | 0.011 | 0.941 |
|  |  | PDQ-39 | 0.111 | 0.444 |
|  |  | BDI | -0.012 | 0.934 |
|  |  | MMSE | -0.050 | 0.718 |
|  |  | LEDD | 0.042 | 0.773 |

LPD, left-onset Parkinson's disease; RPD, right-onset Parkinson's disease; UPDRS-III, Unified Parkinson's Disease Rating Scale motor section; MMSE, Mini-Mental State Examination; PDQ-39, Parkinson's Disease Questionnaire; BDI, Beck Depression Inventory; LEDD, levodopa equivalent daily dose; SMG, supramarginal gyrus; PCUN, precuneus; LING, lingual gyrus; IPL, inferior parietal lobule.
